# Does the left-handed writer in primary school have the same postural and gestural organization as the right-handed for the same quality of graphomotor writing ?

**DOI:** 10.1192/j.eurpsy.2026.11115

**Published:** 2026-07-31

**Authors:** L. Vaivre-Douret, A. Soter, C. Lopez

**Affiliations:** 1 Faculty of Health, Department of Medicine Paris Descartes, University of Paris Cité; 2 Chair of Neurodevelopmental Clinical Phenotyping, Institut Universitaire de France (IUF); 3 Faculty of Medicine, University of Paris-Saclay, UVSQ, Villejuif, Research team “Neurodevelopment and learning disabilities”, Necker-Enfants Malades hospital, INSERM Unit 1018-CESP; 4 Necker Enfants Malades University hospital, AP-HP. Centre; 5 Department of Endocrinology, Necker-Enfants Malades hospital, IMAGINE Institute, Paris, France

## Abstract

**Introduction:**

Writing assessments do not take into account the posturo-gestural organization, and the left-hander is often compared to the right-handed.

**Objectives:**

We aimed to investigate the temporal-spatial quality and posture-gestural of handwriting taking into account the hand laterality of the child.

**Methods:**

84 typically developing children from 1rst to 3 rd grade, with 11% (n=9) of left-handed executed the copy of pre-scriptural drawing of anticlockwise cycloid loops, previously validated as a robust task to predict good handwriting (Lopez & Vaivre-Douret, Children 2023*;* 10, 1512).This task is executed with a digital pen on a specific sheet of paper that allowed spatial-temporal and kinematic measures. All the children were recorded with 2 video-cameras and the gestural and postural aspects analyzed in 2 D reconstruction.

**Results:**

There is an increase in the speed of tracing between 1^rst^ and 3^rd^ grade with more loops without significant difference between right-handed and left-handed children. It is observed from 2^d^ grade that left-handers tilt their heads close to the table (p < 0.01), lift their shoulders (p = 0.01), and have no digital mobility (p = 0.04) while placing the hand that writes above the line.

**Conclusions:**

Regardless of the laterality, the spatial-temporal and kinematics parameters do not differ in the quality. However, the left-handed child uses a posturo-gestural compensation to control the visuo-kinesthetic feedback of the left-to-right draw. Important step to respect for the left-handed child allowing him to access the

automation of the graphic gesture.

**Disclosure of Interest:**

None Declared

